# Supplementary material for: Prolonged Piezo1 Activation Induces Cardiac Arrhythmia
Source: Int J Mol Sci. 2023 Apr 4;24(7):6720. doi: 10.3390/ijms24076720 (PMC10094979; doi:10.3390/ijms24076720)
Supplement: Supplementary file 1 [file ijms-24-06720-s001.zip › figure S1.pdf]

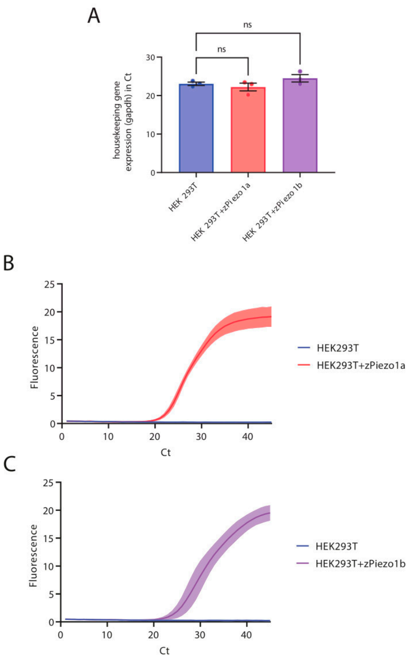

Figure S1. Expression of *piezo1a* and *piezo1b* in transfected HEK293T cells. (**A**) Housekeeping gene expression in non-transfected, (HEK293T) or transfected (HEK293T + zf Piezo1a, HEK293T + zf Piezo1b) cells. Data presented as individual values, mean ± SEM. n=3 for each condition. (**B,C**) Expression of *piezo1a* (**B**) and *piezo1b* (**C**) in transfected compared to non-transfected HEK293T cells. Data presented as average fluorescence curve and the filled area corresponding to the standard deviation. n=3 for each condition. Housekeeping gene expression was statistically analysed using one-way ANOVA.
